# Nicotinamide attenuates the virulence of colorectal cancer-associated *Fusobacterium nucleatum*

**DOI:** 10.1128/iai.00488-25

**Published:** 2026-03-30

**Authors:** Ziyi Yang, Zixue Lei, Qizhao Ma, Jumei Zeng, Mai Xu, Dian Zhang, Pei Xu, Kai Li, Tao Gong, Yuqing Li

**Affiliations:** 1 State Key Laboratory of Oral Diseases & National Center for Stomatology & National Clinical Research Center for Oral Diseases, West China Hospital of Stomatology, Sichuan University 12530, Chengdu, Sichuan, China; 2 West China School of Public Health and West China Fourth Hospital, Sichuan University 12530, Chengdu, China; University of Pennsylvania School of Veterinary Medicine, Philadelphia, Pennsylvania, USA

**Keywords:** *Fusobacterium nucleatum*, nicotinamide, FomA, colorectal cancer

## Abstract

*Fusobacterium nucleatum* contributes to the progression of colorectal cancer (CRC). Nicotinamide (NAM), a safe, cost-effective, and water-soluble form of vitamin B3, has been shown to inhibit the virulence of various microorganisms. However, its effect on *F. nucleatum* pathogenicity remains unclear. In this study, we evaluated the effects of NAM on the pathogenic traits of *F. nucleatum*. A quarter of the minimum inhibitory concentration of NAM (~50 mM) was found to simultaneously inhibit bacterial growth, biofilm formation, and the adhesion and invasion of CRC cells. Transcriptomic analysis of *F. nucleatum* treated at this concentration revealed that 257 genes were upregulated and 316 genes were downregulated. Kyoto Encyclopedia of Genes and Genomes and Gene Ontology functional enrichment analyses indicated that these differentially expressed genes are involved in multiple pathways, including oxidative phosphorylation, biofilm formation, two-component systems, ATP synthesis, and other processes. Furthermore, we showed that NAM, as a class III histone deacetylase inhibitor, could inhibit the deacetylase activity of CobB, thereby increasing the acetylation level of the FomA and reducing its binding ability to CRC cells. In summary, these findings suggest that NAM can attenuate multiple virulence traits of *F. nucleatum* and may serve as a promising *F. nucleatum-*targeted strategy for prevention and treatment of CRC.

## INTRODUCTION

Colorectal cancer (CRC) is a global public health issue with high incidence and mortality rates caused by a combination of genetic and environmental factors (1, 2). In addition to non-genetic factors such as diet and obesity, increasing attention has been focused on the role of microorganisms in promoting the development of CRC (3, 4). The gut microbiota modulate the progression of CRC through the release of different metabolites, proteins, and macromolecules (5). Harmful metabolites including secondary bile acids produced by the gut microbiota accelerate the development of CRC (6). Certain bacterial toxins are involved in the increase of CRC metastasis (7). Moreover, comprehensive CRC analysis revealed consistent gut microbial features across multiple cohorts and their associated serum metabolite profiles (8).

*Fusobacterium nucleatum* is a common opportunistic pathogen and carcinogenic bacterium frequently located in the human oral cavity and gastrointestinal tract (9, 10). It is considered as a key pathogenic factor and microbial biomarker for CRC, promoting its progression and negatively affecting its prognosis (11). This bacterium facilitates tumor development predominantly through virulence factors that enhance its ability to adhere to and invade host cells. The pathogenicity of *F. nucleatum* is largely driven by its surface adhesins, which mediate critical interactions with host cells. Key adhesins include FadA (12), Fap2 (13, 14), RadD (15), and FomA (16). Notably, FadA binds to host E-cadherin, activating β-catenin signaling to directly promote cellular proliferation (12). Similarly, RadD engagement with CD147 initiates a PI3K–AKT–NF–κB–MMP9 cascade that facilitates tumor progression (15). FomA is one of the most abundant outer membrane proteins in *F. nucleatum*, closely related to its adhesion and invasion through the binding to the fragment crystallizable region of human immunoglobulin G (17). In addition, FomA from extracellular vesicles extensively accumulates in CRC tissue and remains on the surface of receptor cells, where it interacts with the fusobacterial surface protein Fn1441 to mediate *F. nucleatum* self-aggregation. This interaction facilitates the translocation of *F. nucleatum* from the oral cavity to the CRC tissue and contributes to the exacerbation of CRC (16).

The extensive research confirming the strong association between *F. nucleatum* and CRC makes clear that the inhibition of *F. nucleatum* virulence may serve as a potential strategy to slow CRC progression (18, 19). Nicotinamide (NAM) is the amide derivative of water-soluble vitamin B3, possessing inhibitory effects on the growth and virulence of various microorganisms (20–22). It not only possesses therapeutic potential in intestinal diseases due to its antimicrobial activity (23) , but also improves disease outcome by attenuating the inflammatory response in colitis animal models (24).

Therefore, this study aimed to evaluate the potential ability of NAM to suppress *F. nucleatum*-mediated CRC progression. We provided an insight into the effect of NAM on *F. nucleatum* virulence by integrating *in vitro* phenotypic assay, transcriptomic profiling, and *in vivo* tumor model. Our results suggest that NAM may serve as a *F. nucleatum*-targeted approach for CRC intervention, which could potentially be translated into clinical applications to improve patient outcomes, especially in cases with high *F. nucleatum* abundance.

## RESULTS

### NAM inhibits the growth and biofilm formation of *F. nucleatum*

**Fig 1:**
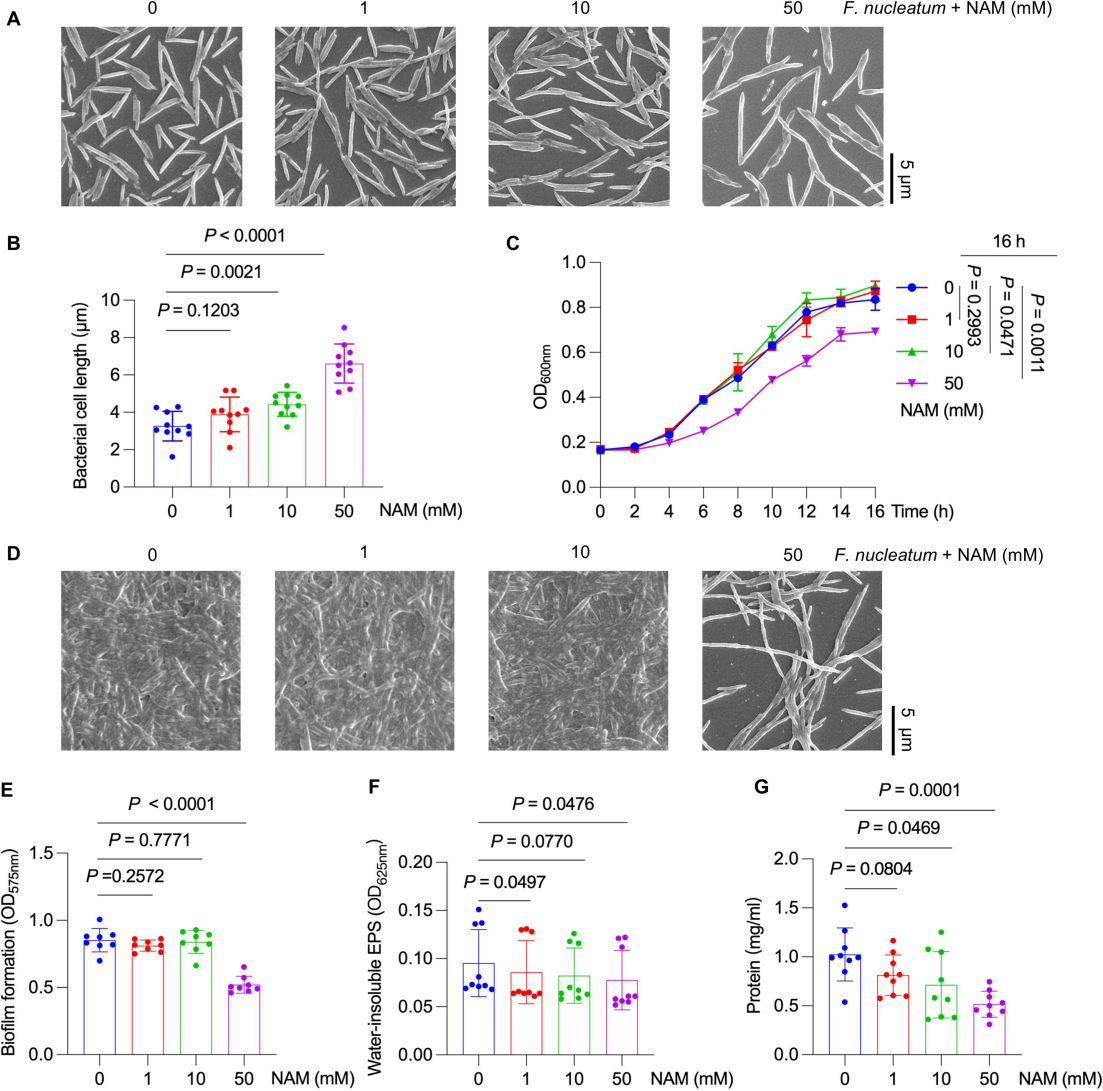
Effect of NAM on bacterial growth and biofilm formation in *F. nucleatum*. (**A**) Representative images of bacterial morphology analyzed by scanning electron microscopy (SEM) at 5,000× magnification. (**B**) Bacterial length from SEM was quantified by ImageJ. Each dot represents one bacterial cell. (**C**) Growth curves were measured under anaerobic conditions for 16 h. (**D**) Representative images of bacterial biofilm structure visualized by SEM at 5,000× magnification. (**E**) Biofilm biomass determined by crystal violet (CV) staining assay. (**F**) Quantification of water-insoluble exopolysaccharide (EPS) content in biofilms. (**G**) Quantification of protein levels in biofilms. The experiment was independently performed three times and each time had two or three biological replicates in **E, F, and G**. Results are presented as mean ± standard deviation (SD), and each dot represents an individual sample in **E, F, and G**. *P* values were determined by unpaired two-tailed Student’s *t*-test in **B, C, E, F**, and **G**.

The effect of NAM on *F. nucleatum* was assessed by measuring the minimum inhibitory concentration (MIC) against *F. nucleatum* using a series of gradient-diluted NAM solutions (0 to 800 mM). Bacterial growth was completely inhibited with a MIC of 200 mM (Fig. S1A and B). Based on this result, NAM concentrations of 0, 1, 10, and 50 mM (1/4 MIC) were selected for subsequent experiments. Bacterial cell length increased in the presence of 10 or 50 mM NAM compared to 0 or 1 mM NAM, as revealed by scanning electron microscopy (SEM) combined with ImageJ-based quantification of bacterial cell length (Fig. 1A and B). The growth curve revealed that 50 mM NAM significantly inhibited the growth of *F. nucleatum*, whereas no significant effect was observed at 0 or 1 mM NAM (Fig. 1C).

Since biofilm formation is an important virulence factor of *F. nucleatum* (9, 25), the effect of NAM on biofilm formation was investigated using SEM and crystal violet (CV) staining. Biofilm formation was significantly inhibited, and sparser biofilm architecture was observed at a concentration of NAM of 50 mM (Fig. 1D and E). Biofilm quantification normalized to planktonic biomass (Fig. S1C) confirmed that additional mechanisms were involved although growth inhibition partially contributed to biofilm reduction. Biofilms are defined as structured microbial communities adherent to surfaces, encapsulated in extracellular polymeric substances composed of exopolysaccharides (EPS), proteins, and other biomolecules (26). The water-insoluble EPS in NAM-treated biofilms was significantly reduced at 1 or 50 mM NAM, with no evident reduction observed at 10 mM compared to the untreated control (Fig. 1F). The protein content in the biofilm matrix was also significantly decreased in the 10 or 50 mM NAM-treated groups compared to the untreated control (Fig. 1G). Collectively, these findings indicated that 50 mM NAM not only altered the morphology and inhibited the growth of *F. nucleatum* but also suppressed biofilm formation by impairing bacterial growth, as well as the production of EPS and proteins.

### NAM suppresses *F. nucleatum* adhesion and invasion, as well as tumor cell proliferation

**Fig 2:**
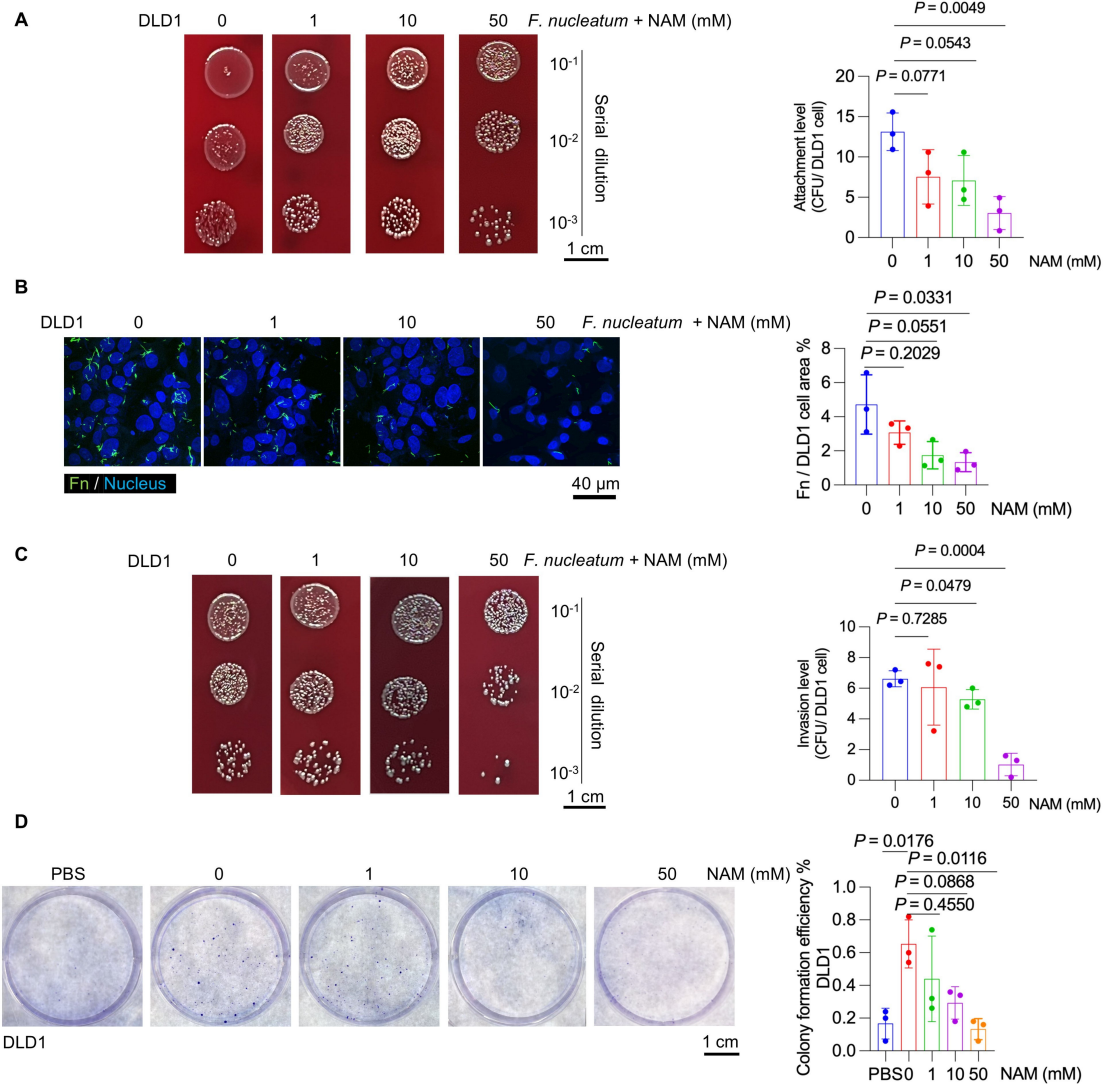
NAM impairs *F. nucleatum* adhesion and invasion, as well as CRC cell proliferation. (**A**) Bacterial attachment to DLD1 cells assessed by CFU assay. Representative images of the attachment to the plates are shown (left), with the corresponding quantification (right). (**B**) Representative fluorescence microscopy images of FITC-labeled (green) *F. nucleatum* or NAM-treated *F. nucleatum* attached to DAPI-stained (blue) DLD1 cells. Quantification of bacterial attachment is shown on the right. (**C**) Invasion of *F. nucleatum* into DLD1 cells evaluated using CFU assay after antibiotic protection. Representative images of the invasion plates (left) and corresponding quantification (right). (**D**) Proliferative response of DLD1 cells to *F. nucleatum* with or without NAM evaluated by plate cloning experiment. Quantification of colony number is shown on the right. The experiment was independently performed at least two times. Results are presented as mean ± SD. Each dot represents an individual sample in** A**, **B**, **C**, and **D**. *P* values were determined by unpaired two-tailed Student’s *t*-test in A, B, and C, one-way analysis of variance (ANOVA) with Tukey’s multiple comparisons test in **D** (PBS: blank control).

The adhesion and invasion of *F. nucleatum* to host cells are important virulence factors contributing to pathogenesis (27, 28). The impact of NAM on the adhesion of *F. nucleatum* was assessed by colony-forming unit (CFU) counting, revealing that 50 mM NAM significantly inhibited the adhesion of *F. nucleatum* to human DLD1 cells compared to other concentrations of NAM (0, 1, and 10 mM) (Fig. 2A). NAM at 50 mM effectively reduced the adhesion of *F. nucleatum* to DLD1 cells compared to 0 mM NAM, as revealed by fluorescence *in situ* hybridization (FISH) (Fig. 2B). Similar results were observed in HCT116 human cells and MC38 mouse cells, with a similar trend of reduced adhesion seen by FISH in MC38 cells, despite it not reaching statistical significance (Fig. S2A through D).

The effect of NAM on the invasion of *F. nucleatum* into host cells was assessed by an antibiotic protection assay to eliminate extracellular bacteria, and intracellular bacteria were quantified by CFU counts collected from lysed host cells. The results demonstrated that 50 mM NAM significantly inhibited the invasion of *F. nucleatum* into DLD1 cells compared to 0 and 1 mM NAM (Fig. 2C), as well as in HCT116 and MC38 cells (Fig. S3A and B), indicating that 50 mM NAM effectively suppressed bacterial invasion in multiple CRC cell lines.

Since *F. nucleatum* promotes CRC cell proliferation (15, 29), the effect of NAM on *F. nucleatum*-induced CRC cell proliferation was evaluated. *F. nucleatum* (without NAM treatment) significantly increased the number and size of colonies formed by DLD1 cells compared to the control group (PBS) (Fig. 2D). However, 50 mM NAM significantly inhibited *F. nucleatum*-induced colony formation (Fig. 2D). Similar inhibitory effect on colony formation was also observed in HCT116 and MC38 cells (Fig. S3C and D). Collectively, these findings demonstrated that 50 mM NAM inhibited key virulence properties of *F. nucleatum*, including its ability to attach, invade, and promote proliferation of CRC cells.

### Transcriptome analysis of NAM-treated *F. nucleatum*

**Fig 3:**
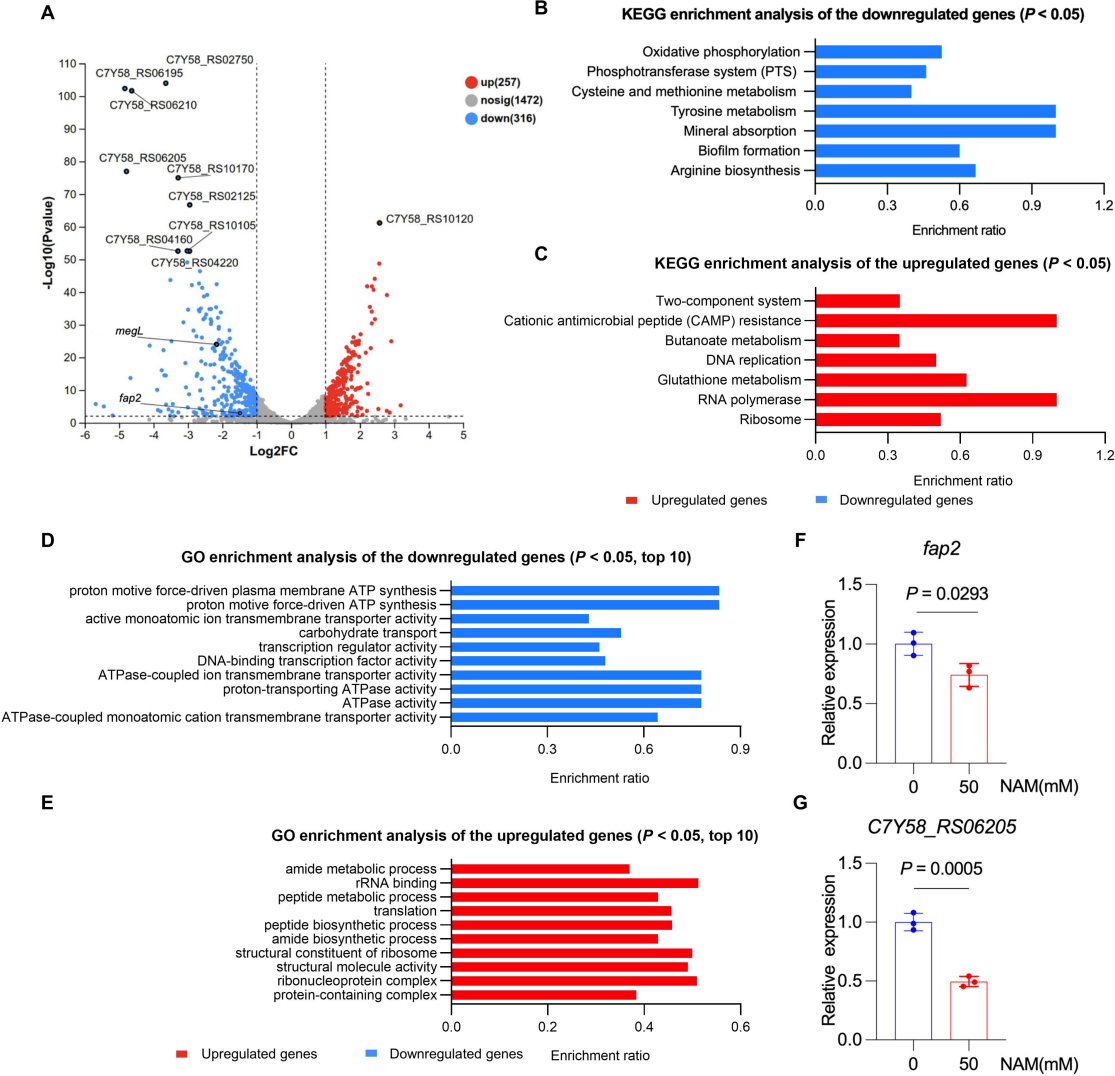
Transcriptomic analysis of NAM-treated *F. nucleatum*. (**A**) Volcano plot showing differentially expressed genes (DEGs) in *F. nucleatum* after the treatment with 50 mM NAM. (**B, C**) KEGG pathway enrichment analysis of downregulated (**B**) and upregulated (**C**) genes. (**D, E**) GO enrichment analysis of downregulated (**D**) and upregulated (**E**) genes. RT-qPCR was used to analyze the expression levels of *fap2* (F）and *C7Y58_RS06205* (**G**) in *F. nucleatum* under 0 or 50 mM NAM treatment. Differential expression analysis was performed using DESeq2 with BH-adjusted *P* values. KEGG pathway enrichment significance was evaluated using Fisher’s exact test with BH correction, while GO enrichment significance was assessed using Fisher’s exact test with Bonferroni correction. Results are presented as mean ± SD of three biological replicates, and each dot represents an individual sample in **F and G**. *P* values were determined by an unpaired two-tailed Student’s *t*-test in **F and G**. Cut-off indicates twofold change (FC) > 2 in expression and *P* < 0.01 as described in the Materials and Methods.

The impact of NAM on *F. nucleatum* gene expression was investigated by the transcriptome analysis of bacteria cultured with or without 50 mM NAM. Differential gene expression analysis revealed 316 significantly downregulated genes and 257 significantly upregulated genes in the NAM-treated group compared to the untreated control (Fig. 3A).

The biological implications of these transcriptional changes were assessed by the enrichment analysis of the differentially expressed genes (DEGs). Kyoto Encyclopedia of Genes and Genomes (KEGG) enrichment analysis performed using KOBAS 2.0 revealed that downregulated genes were significantly enriched in multiple pathways, including oxidative phosphorylation, phosphotransferase system (PTS), cysteine and methionine metabolism, tyrosine metabolism, mineral absorption, biofilm formation, and arginine biosynthesis (Fig. 3B). The upregulated genes were enriched in multiple pathways, including two-component system, cationic antimicrobial peptide (CAMP) resistance, butanoate metabolism, DNA replication, glutathione metabolism, RNA polymerase, and ribosome pathways (Fig. 3C).

Gene Ontology (GO) enrichment analysis performed using GOATOOLS further demonstrated that the most significantly downregulated GO terms included proton motive force-driven plasma membrane ATP synthesis, proton motive force-driven ATP synthesis, ATPase-coupled ion transmembrane transporter activity, proton-transporting ATPase activity, and ATPase activity (Fig. 3D). The most upregulated GO terms included rRNA binding, structural constituent of ribosome, structural molecule activity, and ribonucleoprotein complex (Fig. 3E).

In summary, NAM treatment induced extensive reprogramming of the *F. nucleatum* transcriptome. The transcriptome results showed that the core pathways responsible for fundamental bacterial life activities and colonization (30), including oxidative phosphorylation, phosphotransferase system (PTS), and ATP synthesis (Fig. 3B and D), were markedly downregulated. The upregulated pathways, including the two-component system, CAMP resistance, and peptide biosynthetic process, may enable *F. nucleatum* to better respond to NAM stress (Fig. 3C and E). Notably, the downregulated biofilm formation pathway may lead to a reduction in biofilm biomass (Fig. 1E and 3B). These correlative data lay a foundation for elucidating NAM’s antibacterial and anti-biofilm effects.

### NAM increases FomA acetylation through CobB inhibition and impairs its adhesion to CRC cells

NAM is a competitive inhibitor of the NAD^+^-dependent sirtuin-type deacetylase, CobB (31). CobB mediates bacterial post-translational regulation through site deacetylation of lysine residues on target proteins (32). FomA is the main outer membrane protein in *F. nucleatum* and is well characterized as a critical adhesin involved in bacterial attachment and biofilm formation (16, 33). Given that transcriptomic profiling revealed no significant change in *fomA* expression following treatment with 50 mM NAM, we hypothesized that NAM impairs adhesion of *F. nucleatum* by modulating the acetylation status of FomA rather than its gene expression.

**Fig 4:**
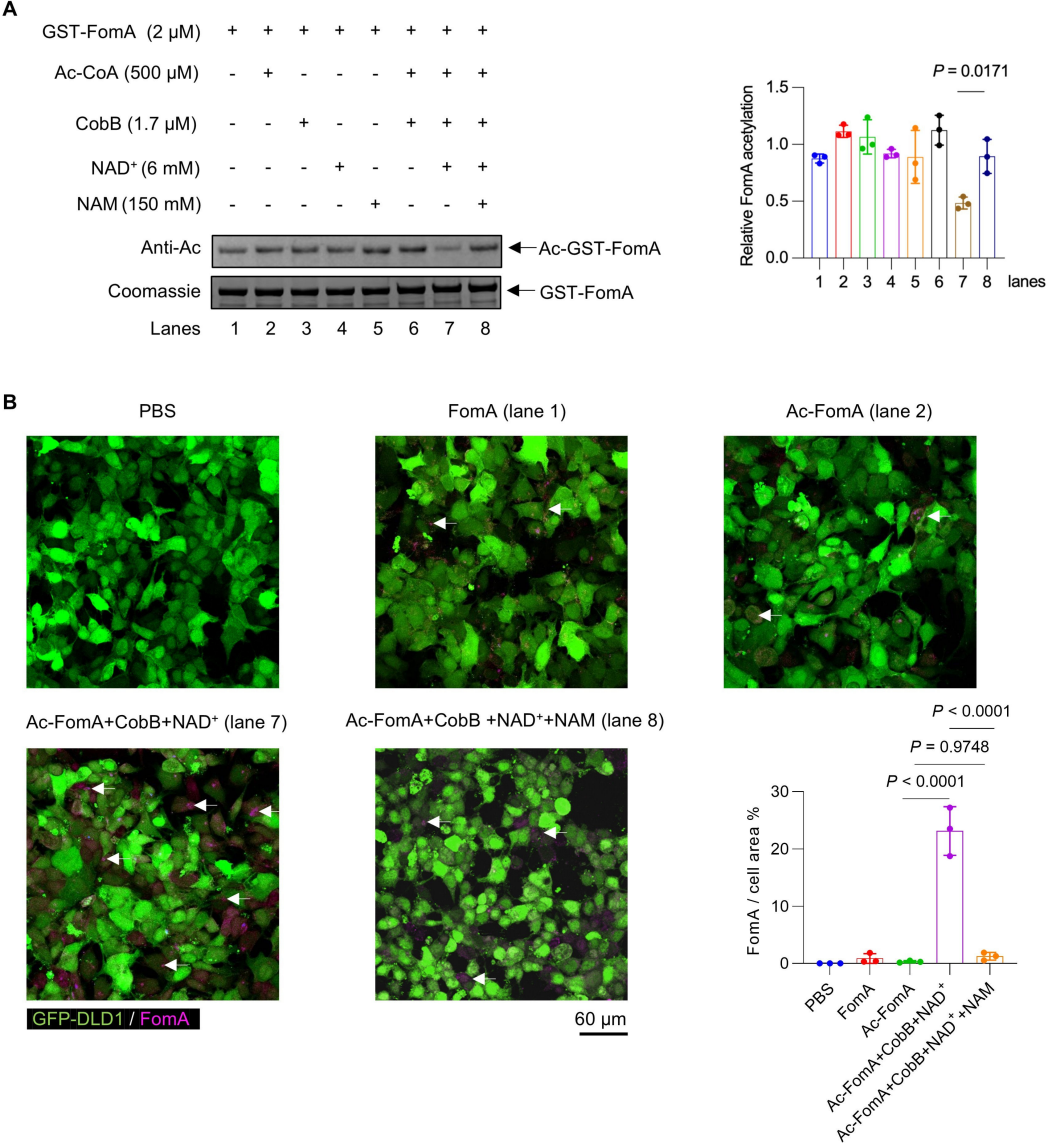
Effect of NAM inhibition of CobB on FomA acetylation and DLD1 cell attachment. (**A**) Coomassie staining and anti-acetyl lysine western blotting were performed on recombinant FomA incubated with acetyl-CoA (acetyl donor) for 3 h at 37°C. Quantification of FomA and Ac-FomA is shown on the right. (**B**) Immunofluorescence analysis performed to assess the binding of Alexa Fluor 594-labeled (pink) FomA or Ac-FomA to GFP-labeled (green) DLD1 cells. GFP-labeled DLD1 cells treated with FomA under five conditions: (1) cells alone (PBS), (2) untreated FomA, (3) acetylated FomA, (4) CobB-deacetylated FomA, and (5) FomA co-treated with CobB and NAM. White arrows indicate the localization of FomA and Ac-FomA on the host cell surface. Quantification of FomA and Ac-FomA binding is shown on the bottom right corner. The experiment was independently performed at least two times. Results are presented as mean ± SD. Each dot represents an individual sample in A and B. *P* values were determined by one-way ANOVA with Tukey’s multiple comparisons test in A and B (Ac-FomA: acetylated FomA).

To test this hypothesis, the effect of NAM on the acetylation level of FomA was evaluated. We found that FomA was acetylated when purified from *Escherichia coli* (Fig. 4A, lane 1). The addition of Ac-CoA, CobB, NAD^+^, or NAM alone had no significant effect on the acetylation level of FomA (Fig. 4A, lanes 2–5). CobB decreased the acetylation level of FomA in the presence of NAD^+^ (Fig. 4A, lanes 6 and 7). On the contrary, the addition of NAM restored the acetylation level of FomA (Fig. 4A, lanes 7 and 8). These data suggest that NAM may inhibit CobB activity to increase the acetylation level of FomA.

To investigate the effect of NAM on the adhesion of FomA to host cells, we co-cultured GFP-DLD1 cells with FomA and performed an immunofluorescence staining. The results showed that the amount of FomA adhering to DLD1 cells in the “Ac-FomA+CobB + NAD^+^” group was higher than that in the “FomA” and “Ac-FomA” groups (Fig. 4B). However, the addition of NAM significantly inhibited the adhesion of FomA to DLD1 cells, as shown in “Ac-FomA+CobB + NAD^+^+NAM” group (Fig. 4B). Collectively, these findings demonstrate that NAM may increase FomA acetylation by inhibiting CobB, thereby impairing the adhesion of FomA to host cells.

### NAM attenuates *F. nucleatum*-induced CRC tumorigenesis in mice

**Fig 5:**
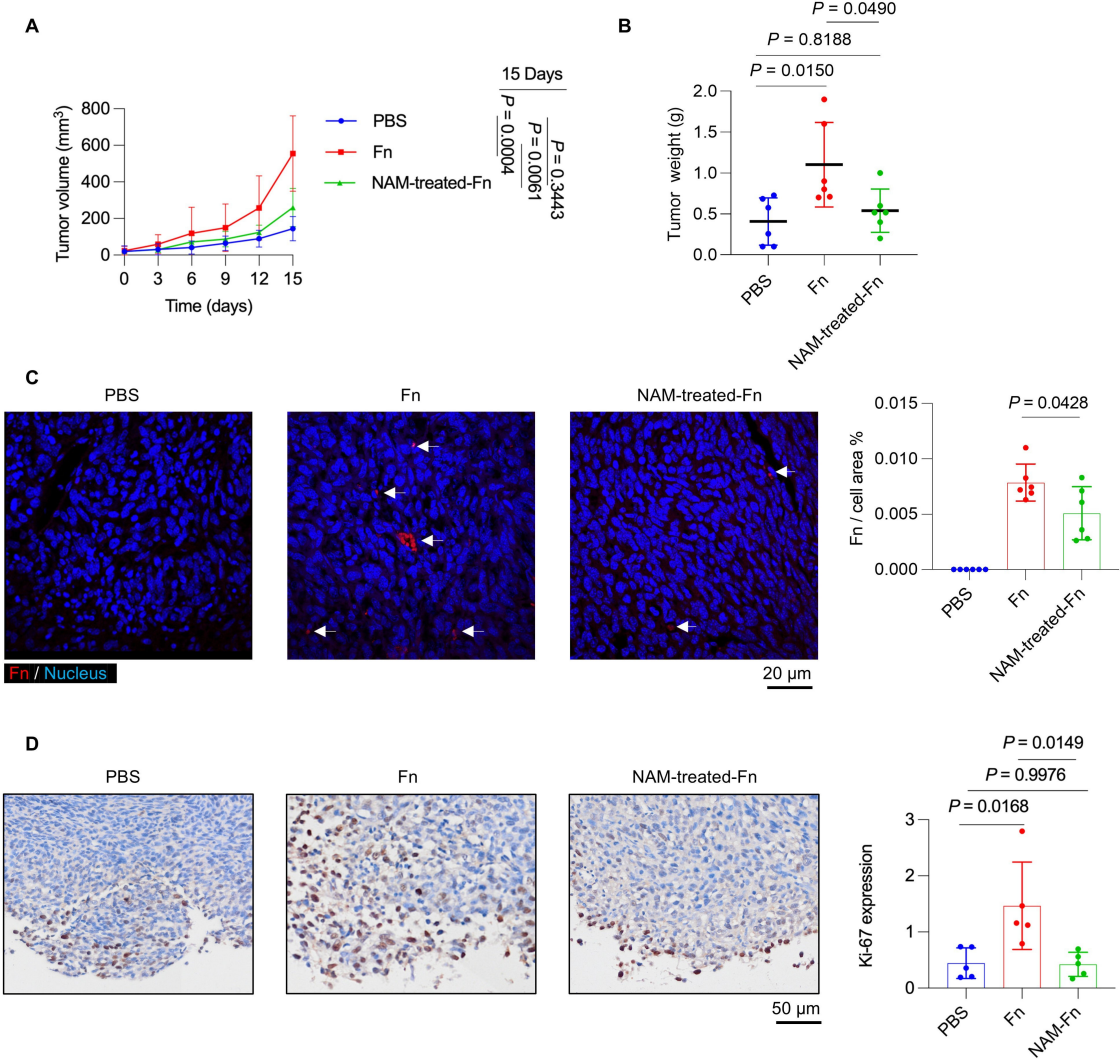
NAM attenuates colonic tumorigenesis in *F. nucleatum*-induced C57BL/6 mice. (**A, B**) Effect of peritumoral injection of the indicated bacterial treatments on MC38 subcutaneous tumors in C57BL/6 mice assessed by measuring tumor volume (mm^3^) (**A**) and weight (**B**). (**C**) *F. nucleatum* in colonic tumors of C57BL/6 mice by FISH, with corresponding quantification (right). *F. nucleatum* and NAM-treated *F. nucleatum* in tumors are indicated by white arrows. (**D**) Ki-67 positive cells in colon tumors by IHC and quantification using ImageJ (right). *n* = 6 biological replicates in each group from two independent experiments. Results are presented as mean ± SD. Each dot represents an individual sample in **A, B, C, and D**. *P* values were determined by one-way ANOVA with Tukey’s multiple comparisons test in **A, B, and D** and unpaired two-tailed Student’s *t*-test in **C** (Fn: *F. nucleatum*).

The impact of NAM on *F. nucleatum*-mediated CRC tumor progression was established using a subcutaneous MC38 tumor model developed in C57BL/6 mice. Both tumor volume and weight were significantly reduced in the “NAM-treated Fn” group compared to the “Fn” group (Fig. 5A and B). NAM inhibited the colonization of *F. nucleatum* in mouse tumor tissues, as revealed by FISH staining (Fig. 5C). Ki-67 is a well-established nuclear marker of cell proliferation in CRC. Immunohistochemistry of Ki-67 in mouse tumor tissues confirmed that NAM attenuated *F. nucleatum*-induced tumor growth (Fig. 5D). Collectively, these results demonstrated that NAM treatment reduced the tumor-promoting effects of *F. nucleatum in vivo* through the suppression of bacterial colonization and tumor cell proliferation.

## DISCUSSION

*F. nucleatum* has emerged as a key contributor to CRC progression and treatment resistance (11) and is frequently enriched in CRC tissues (19). In this study, we demonstrate that NAM reduces the growth of *F. nucleatum* and attenuates its adhesion and invasion. Taken together with molecular and functional evidence, our results suggest that NAM may attenuate *F. nucleatum* host colonization by modulating NAD⁺-dependent deacetylation CobB and altering the post-translational modification status of adhesion-related proteins. Both *in vitro* and *in vivo* experiments further substantiate that NAM effectively ameliorates *F. nucleatum*-driven CRC progression.

We first evaluated the impact of NAM on the core physiological functions of *F. nucleatum*. NAM significantly suppressed both the growth and biofilm formation of *F. nucleatum*, with an effective concentration substantially lower than that required to target other oral microorganisms (20, 21). This result is consistent with the previously reported metabolic regulatory roles of NAM in other bacterial species and is validated here for the first time in *F. nucleatum*. Transcriptomic profiling revealed that NAM treatment significantly downregulated genes involved in carbohydrate transport and oxidative phosphorylation, potentially limiting energy acquisition and biosynthetic capacity in *F. nucleatum* (34). The latter pathway is important for *F. nucleatum* energy metabolism, as the bacterium relies on oxidative phosphorylation for efficient ATP generation (35). Although direct measurement of oxidative phosphorylation activity was not conducted, the downregulation of this pathway may correlate with the reduced bacterial growth rate (Fig. 1C), which is in line with findings in other pathogens where impaired oxidative phosphorylation leads to compromised proliferation (36, 37). In addition, the upregulation of glutathione metabolism-related genes suggested an increased oxidative stress following NAM exposure, indicating a compensatory mechanism aimed at restoring redox balance (38). These findings demonstrated that NAM disrupted an energy generation and redox balance that ultimately undermined *F. nucleatum* fitness and proliferation.

The key carcinogenic role of bacteria in the tumor microenvironment lies in their adhesion to host cells (39). Previous studies have demonstrated that Fap2 encodes an adhesin that facilitates immune evasion by binding to host inhibitory receptors such as CEA cell adhesion molecule 1 (CEACAM1), TIGIT, and Gal GalNac (13, 14), while the fructose phosphotransferase system (PTS), beyond its role in carbohydrate uptake, is involved in the regulation of biofilm formation and bacterial adhesion (40, 41). In line with these findings, our transcriptomic analysis revealed that NAM treatment significantly downregulated the expression of the virulence factor *fap2* and reduced the transcription of a gene associated with the fructose PTS (*C7Y58_RS06205*) (Fig. 3A, F and G), suggesting that NAM may attenuate *F. nucleatum* adhesion and invasion through multiple regulatory pathways. These findings further underscore the role of adhesion-related pathways in *F. nucleatum* virulence and indicate that NAM, as an exogenous metabolic molecule, may have potential involvement in modulating these pathogenic processes.

Protein acetylation has emerged as an important regulatory mechanism governing pathogen virulence and host–microbe interactions (42, 43). The deletion of *cobB* markedly impairs the intestinal colonization of enterohemorrhagic *E. coli* (32). The acetylation of pyruvate kinase mediated by acetyltransferase ActA negatively regulates the oxidative stress adaptability of *Streptococcus mutans* (44). The deletion of a NAD^+^-dependent deacetylase in *Mycobacterium tuberculosis* leads to impaired biofilm formation (45). The regulation of acetylation modification also exists in the tumor microenvironment. The combined treatment of lycorine and NAM enhances the acetylation of isocitrate dehydrogenase, an essential enzyme involved in energy metabolism, and induces mitochondrial dynamic imbalance in CRC cells (46). Our study provides corroborating evidence in *F. nucleatum* that NAM can alter the acetylation level of FomA by inhibiting CobB activity and affecting its adhesive ability. Given the role of FomA in bacterial aggregation and immune modulation, this observation demonstrated a functional connection between CobB activity and FomA-mediated virulence in *F. nucleatum*, highlighting that CobB-mediated regulation of adhesins might represent a broadly conserved strategy among pathogens and a promising approach for therapeutic intervention. Notably, the effects of NAM could also attenuate *F. nucleatum* pathogenicity by suppressing *fap2* expression. However, it is difficult for us to clearly distinguish whether *fap2* expression downregulation or FomA acetylation is responsible for *F. nucleatum* attachment and invasion experimentally; both may act together.

Consistent with these *in vitro* results and mechanisms, our subcutaneous tumor model demonstrated that NAM treatment significantly attenuated *F. nucleatum*-driven CRC progression *in vivo*, as shown by the reduced tumor volume, lower bacterial colonization in the tumor, and decreased expression of the proliferation marker Ki-67. Taken together, our study systematically revealed that NAM inhibited *F. nucleatum*-mediated CRC progression by suppressing its adhesion and invasion. This research not only increased the number of effective non-antibiotic antimicrobial strategies but also highlighted the regulation of bacterial virulence through a post-translational intervention, thereby providing new approaches for gut microbiota therapy.

Despite the significant progress, several questions remain unresolved. The transcriptomic changes observed, including alterations in metabolic pathways such as oxidative phosphorylation and arginine biosynthesis, still require functional validation through integrated metabolomic studies to confirm their phenotypic impact. And then, genetic validation of CobB’s causal role in the NAM-mediated effects is currently limited because construction of *cobB* deletion and complemented strains in *F. nucleatum* ATCC 25586 remains technically challenging (47, 48). Moreover, although our data indicate that NAM treatment is associated with CobB inhibition and increased FomA acetylation, the precise molecular mechanism by which acetylation modulates FomA binding to host-cell receptors remains to be elucidated. Future studies will employ alternative genetic strategies such as CRISPR interference (49) to establish causal genetic evidence linking cobB to FomA acetylation, identify the specific lysine residues of FomA that undergo acetylation, and determine how this post-translational modification affects FomA’s structural conformation and its interactions with host cell receptors. Furthermore, the subcutaneous tumor model, though informative, does not fully capture the complexity of the colorectal microenvironment, employing orthotopic models would enhance physiological relevance. Ultimately, despite the safety and tolerability of NAM, as a water-soluble vitamin B3 (50, 51), critical clinical parameters in CRC patients, such as pharmacokinetic profiles, optimal administration routes, and tissue-specific accumulation, require further investigation through larger-scale clinical trials. Future research should also explore the synergistic effects of NAM when combined with other targeted or immunotherapeutic agents, which will provide a robust foundation for its clinical translation.

## MATERIALS AND METHODS

### Bacterial culture

*F. nucleatum *from the American Type Culture Collection (ATCC) 25586 was provided by the State Key Laboratory of Oral Diseases, Sichuan University. It was cultured in Columbia broth (Solarbio, China) supplemented with hemin (5 μg/mL) and menadione (0.5 μg/mL) (J&K Scientific, China) (CBHK) and incubated at 37°C under anaerobic conditions (90% N_2_, 5% CO_2_, and 5% H_2_). The OD_600 nm_ of the bacterial culture was measured to evaluate its growth, while CFU counting was performed on Columbia blood agar (HuanKai Microbial, China).

### Cell culture

The human and mouse CRC cell lines DLD1, HCT116, and MC38 were obtained from ATCC and Kerafast (USA). Cells were cultured in DMEM (Gibco, USA) supplemented with 10% fetal bovine serum (FBS, Every Green, China) and 1% penicillin-streptomycin (Abmole, USA) and incubated at 37°C in a humidified 5% CO_2_. GFP‑DLD1 cells were generated as described in a previous research (16), using lentiviral transduction with 8 µg/mL polybrene (Sigma, USA) and 1 µg/mL puromycin selection for GFP‑DLD1.

### MIC assay and bacterial treatment

NAM powder (Solarbio, China) was first dissolved in sterile double-distilled water to obtain a 2 M stock solution, which was sterilized by filtration and stored at –20 °C. MIC of NAM against *F. nucleatum* was assessed by the microdilution method. NAM solutions ranging from 0 to 800 mM were used, and bacterial growth was assessed after 24 h. Overnight *F. nucleatum* culture was diluted in fresh CBHK to an initial OD_600 nm_ of 0.5. NAM stock was diluted with fresh CBHK medium until reaching the final concentrations of 1, 10, and 50 mM, which were added to the cultures. Cultures were incubated at 37 °C for 24 h under anaerobic condition for further analyses.

### Growth curves

Overnight *F. nucleatum* culture was diluted in fresh CBHK to an initial OD_600 nm_ of 0.1, followed by the addition of NAM solution at final concentrations of 1, 10, and 50 mM. Cell culture was incubated under anaerobic conditions at 37°C in a 96-well plate for 16 h, with OD_600 nm_ measured every 2 h to generate the growth curve.

### SEM of biofilms

Biofilm structure and bacterial morphology were analyzed using SEM. Biofilm cultivation was performed based on previous studies with slight modifications (52). *F. nucleatum* was seeded in 24-well plates with NAM at concentrations of 1, 10, and 50 mM and incubated under anaerobic conditions for 36 h, while a NAM-free group was used as the control. Next, biofilms were rinsed two times with PBS and fixed overnight in 2.5% glutaraldehyde at 4°C (53). The biofilms were rinsed again two times with PBS and sequentially dehydrated with ethanol (30%, 40%, 50%, 60%, 70%, 80%, 85%, 90%, 95%, 100%, 15 min per step). Samples were transferred to 100% amyl acetate for 25 min, then subjected to critical point drying with CO_2_. Dried specimens were mounted on aluminum stubs using conductive carbon tape and sputter coated with a gold layer. Finally, samples were examined by SEM (FEI, Hillsboro, OR, USA).

### Crystal violet assay, quantification of the water-insoluble EPS and proteins

Biofilm biomass was assessed using the CV staining method (53). Diluted bacterial strains were seeded in 48-well plates and incubated for 36 h under anaerobic conditions. Next, the growth medium was carefully removed, and biofilms were washed two times with PBS before being stained with 0.1% CV for 5 min. The wells were gently rinsed three times with PBS, followed by the addition of 95% ethanol and incubated for 30 min. Absorbance was measured at OD_575 nm_ using a spectrophotometer (Thermo Scientific, USA). The quantification of water-insoluble EPS (53) and proteins (54) was adapted from previous studies. Proteins were extracted from biofilms using radioimmunoprecipitation (RIPA) assay buffer with a protease inhibitor cocktail (Abmole, USA), and protein concentration was determined by the bicinchoninic acid assay using bovine serum albumin standard (BSA, Beyotime, China).

### Cell proliferation, attachment, and invasion assays

Before co-culture, bacteria were pre-treated with 1, 10, and 50 mM NAM for 24 h, while untreated bacteria were used as blank control. After treatment, bacterial pellets were washed two times with DMEM supplemented with 10% FBS and resuspended in DMEM supplemented with 10% FBS. All bacterial suspensions were re-adjusted to an OD_600 nm_ of 0.5 using the same medium. As regards colony formation, cells in 24-well plates were treated with *F. nucleatum* at MOI = 100 at 37°C for 2 h. And then, *F. nucleatum*-treated cells were detached using trypsin until reaching a single-cell suspension and seeded at low density (5,000 DLD1 cells/well, 4,500 HCT116 cells/well, and 800 MC38 cells/well) in 6-well plates at 37°C for about 2 weeks when a discrete number of colonies was formed. Cells were fixed with 4% paraformaldehyde (PFA) for 30 min. Wells were washed three times with PBS, stained with 0.01% CV for 20 min, and washed three other times with PBS to remove the dye in excess. Colonies containing more than 50 cells were counted.

Attachment and invasion assays were carried out as previously described (15). Briefly, cells in 24-well plates were treated with *F. nucleatum* at MOI = 100 at 37°C for 2 h. As regards the invasion assay, monolayers were washed and treated with metronidazole (200 µg/mL, Abmole, USA) and incubated at 37°C for 1 h to remove extracellular bacteria. Cells were lysed, and serial dilutions of the lysate were plated on Columbia blood agar to count intracellular colony-forming units. Attachment was assessed in the same manner but without antibiotic treatment, thereby quantifying the total cell-associated bacteria.

### RNA-sequencing for transcriptome analysis

*F. nucleatum* with or without 50 mM NAM was diluted 1:10 using fresh CBHK medium and incubated at 37°C for 24 h. Total RNA was extracted using the cetyltrimethylammonium bromide (CTAB) method, ribosomal RNA (rRNA) was removed using the rRNA Depletion Kit (RiboCop, USA) for mixed bacterial samples, and poly(A)–cDNA libraries were constructed with the TruSeq Stranded mRNA Library Prep Kit (Illumina, USA). Libraries were sequenced on a Novaseq Xplus platform (Illumina Inc, USA), and DEG analysis was performed using DESeq2. Genes with a fold-change (FC) > 2.0 and *P* < 0.01 were considered statistically significant (21).

### RNA extraction and qRT-PCR

Bacteria were pre-treated with 50 mM NAM for 24 h, while untreated bacteria were used as blank control. After treatment, bacterial pellets were washed three times with PBS. First, total RNA was extracted and purified using the Total RNA Isolation Kit V2 (Vazyme, China) according to the manufacturer’s instructions. RNA (1 mg) was reverse-transcribed to cDNA using a cDNA synthesis kit (TaKaRa, Japan). Then, quantitative real-time PCR (qRT-PCR) was performed using SYBR green master mix on a LightCycler 480 Real-Time PCR system (Roche, Switzerland). The relative expression levels of genes were calculated using the 2^−ΔΔCt^ method with values normalized to the reference gene 5S rRNA.

### Purification of recombinant FomA and CobB

GST6His-tagged FomA (FomA) was purified as described in a previous research (16). The coding region of ATCC 25586 *fomA* (FN1859), along with GST and 6His-tag coding sequences conjugated to its 3′ end, was cloned into pET-41a(+). The resulting vector was transformed into *E. coli* BL21 (DE3) (Takara, China). The GST-6His-FomA protein was purified using a standard protocol using Ni^2+^ affinity columns (Beyotime, China).

*cobB* was amplified from *F. nucleatum* genomic DNA by PCR and purified. The products were digested by *Nco* I and *Xho* I and then cloned into the expression vector pET28a(+) (Novagen, Germany) with an N-terminal fusion of a 6 × His tag. Next, the reconstructed plasmids were transformed into *E. coli* BL21(DE3) (Tsingke, China). Then, the protein was purified. First, the overnight culture of the transformant was diluted 1:100 with fresh LB medium containing 50 μg/mL kanamycin until an OD_600 nm_ of 0.6 was achieved. After further growth with 1 mM isopropyl-β-D-thiogalactopyranoside at 16°C overnight to induce protein expression, the cell pellets were harvested and lysed by the bead-beating method. Then, the recombinant CobB was purified using a His-tagged protein purification kit (Beyotime, China) according to the manufacturer’s instructions and was concentrated from the cell debris using a 10 kDa MWKO ultrafiltration (Millipore Amincon, Germany). The purified FomA and CobB were confirmed by SDS-PAGE and stored at −80°C for further use.

### Western blotting

Western blotting was performed as previously described (44). Protein concentration was determined using the BSA protein assay kit (Beyotime, China), following the manufacturer’s instructions. Equal amounts of protein were mixed with SDS sample buffer (Beyotime, China), 99°C for 10 min, and separated using 4%–20% SDS-PAGE (140 V) (Yeasen, China). The polypeptides were transferred by electrophoresis into a polyvinylidene difluoride (PVDF) membrane blocked in 5% (wt/vol) nonfat dry milk at room temperature (RT) for 1 h. Then, the membrane was incubated with anti-acetyl lysine antibody diluted 1:1,000 in TBST (Solarbio, China) and incubated at 4°C overnight. After five rounds of washing with TBST, the anti-acetyl lysine membrane was incubated with HRP-conjugated goat anti-mouse secondary antibody at a 1:20,000 dilution in TBST at RT for 2 h. The membrane was developed using the immobilon Western Chemiluminescent HRP substrate kit (Yeasen, China).

### *In vitro* acetylation analysis

*In vitro* acetylation analysis was performed as previously described (44) with minor modifications. Briefly, the purified GST-FomA was incubated with acetyl-CoA in a total reaction volume of 20 μL containing 100 mM Tris-HCl (pH 8.0), 150 mM NaCl, and 10 mM MgCl_2_ in the presence or absence of CobB or NAM. The reaction was performed at 37°C for 3 h and was terminated by 99°C in SDS sample buffer. Then, the samples were analyzed by SDS-PAGE and western blotting.

### Immunofluorescence staining and analysis

Immunofluorescence was performed according to a previous study with minor modifications (55). Five experimental groups were analyzed: (i) untreated cells (control), (ii) FomA-treated cells, (iii) acetylated FomA (Ac-FomA)-treated cells, (iv) CobB-treated-Ac-FomA cells, and (v) NAM-treated-Ac-FomA cells. Overnight-treated cells were centrifuged, fixed with 4% PFA, and incubated at 4°C for 30 min. Fixed samples were washed three times with PBS, blocked with 5% BSA (BioFroxx, Germany) at RT for 1 h, and incubated with homemade anti-FomA antibody (1:500) at 37°C for 2 h. Samples were washed with TBST, exposed to Alexa Fluor 594-conjugated anti-rabbit secondary antibody (1:200, Elabscience, China) at 37°C for 1 h, followed by nuclear counterstaining with DAPI (Solarbio, China), and observed under a confocal microscopy using a FLUOVIEW FV3000 microscope (Olympus). FomA signal intensity was quantified by ImageJ software as the percentage of fluorescence-positive area relative to GFP-defined cellular regions.

### FISH and IHC

FISH was performed as previously described (16). Samples underwent ethanol dehydration (50%, 80%, 100%), followed by hybridization with an Alexa Fluor 488-conjugated *F. nucleatum*-specific probe (5′-GGCTTCCCCATCGGCATT-3′, 200 nM) in 20% formamide buffer (46°C and 90 min, Yuanye, China). Bacterial abundance was quantified as the percentage of probe-positive areas (ImageJ) relative to *F. nucleatum*/DAPI signals. CRC tissue sections were treated with 3% H_2_O_2_ (10 min), blocked with goat serum (37°C, 30 min), incubated with anti-Ki-67 antibody (Abcam, UK) overnight (4°C) followed by the treatment with biotin-HRP secondary antibody (37°C, 30 min, Abcam, UK). Signals were developed using diaminobenzidine (DAB), and the tissue was counterstained with hematoxylin.

### Animal study

Male C57BL/6J mice (6 weeks old, *n* = 6 per group) (56) were treated with a subcutaneous injection of 5 × 10^5^ MC38 CRC cells resuspended in 200 μL sterile PBS into the right scapular region. Mice were randomly divided into groups after 7 days of implantation to receive the following peritumoral injections every 3 days: (i) PBS, (ii) *F. nucleatum* (1 × 10^6^ CFU in PBS), or (iii) *F. nucleatum* pre-treated with 50 mM NAM during anaerobic *in vitro* culture (37°C, 24 h) and subsequently washed three times with PBS to remove residual NAM before being resuspended in sterile PBS at a concentration of 1 × 10^6^ CFU. All mice were euthanized after receiving the five treatments. Tumor growth was monitored every 3 days using a digital caliper, with volume calculated as width^2^ × length/2.

### Statistical analysis

Statistical analysis was performed using Prism 10.0 (GraphPad Software Inc, San Diego, CA, USA). All data were first subjected to Kolmogorov-Smirnov test or Shapiro-Wilk test for normal distribution and to Brown-Forsythe test for homogeneity of variance. Data that passed these two tests were analyzed by unpaired *t-*test or one-way analysis of variance (ANOVA) with the Tukey method as the post hoc test. All experiments were performed in two or three biological replicates per condition and were reproduced at least two times. Results were expressed as mean ± standard deviation (SD). A value of *P* < 0.05 was considered statistically significant.

## Data Availability

The raw data from the transcriptomic sequencing analysis of F. nucleatum ATCC 25586 treated with or without 50 mM NAM have been deposited in the NCBI Sequence Read Archive (SRA) database under BioProject accession number PRJNA1320742.

## SUPPLEMENTAL MATERIAL

Fig. S1 iai.00488-25-s0001.tiff Effect of NAM on *F. nucleatum* growth and biofilm formation. (A) Minimum inhibitory concentration (MIC) was performed in Columbia broth supplemented with 5 μg/mL hemin and 0.5 μg/mL menadione (CBHK). Bottom labels denote NAM concentrations and the acronym CBHK stands for blank control. (B) MIC for untreated *F. nucleatum* and NAM-treated *F. nucleatum* was quantified based on OD_600 nm_ values. (C) Biofilm biomass was quantified by CV staining and normalized to equivalent bacterial density. The experiment was independently performed at least two times. Results are presented as mean ± SD. Each dot represents an individual sample in B and C. *P* values were determined by unpaired two-tailed Student’s *t*-test against the untreated control group in B and C (ns, *P* > 0.05; ***P* < 0.001; *****P* < 0.0001).

Fig. S2 iai.00488-25-s0002.jpg NAM affects *F. nucleatum* adhesion to CRC cells.(A, B) Effect of NAM on *F. nucleatum* attachment to HCT116 (A) or MC38 (B) cells evaluated using CFU assay. (C, D) Representative fluorescence microscopy images showing FITC-labeled (green) *F. nucleatum* and DAPI-stained (blue) HCT116 (C) and MC38 (D) cells. Quantitative analysis of bacterial adhesion is shown on the right for each panel. The experiment was independently performed at least two times. Results are presented as mean ± SD. Each dot represents an individual sample in A, B, C, and D. *P* values were determined by unpaired two-tailed Student’s *t*-test in A, B, C, and D.

Fig. S3 iai.00488-25-s0003.jpg NAM affects *F. nucleatum* invasion and CRC cell proliferation.(A, B) Effect of NAM on the invasion of *F. nucleatum* into HCT116 (A) or MC38 (B) cells evaluated using CFU assay. (C, D) Proliferation of HCT116 (C) and MC38 (D) cells in response to NAM-treated *F. nucleatum* were assessed using plate cloning experiment. Quantification of invading *F. nucleatum* and cell proliferation are shown on the right for each panel. The experiment was independently performed at least two times. Results are presented as mean ± SD. Each dot represents an individual sample in A, B, C, and D. *P* values were determined by unpaired two-tailed Student’s *t*-test in A and B, and one-way ANOVA with Tukey’s multiple comparisons test in C and D.

Table S1 iai.00488-25-s0004.xlsx Differentially expressed *F. nucleatum* genes in response to 50 mM NAM.

ASM does not own the copyrights to Supplemental Material that may be linked to, or accessed through, an article. The authors have granted ASM a non-exclusive, world-wide license to publish the Supplemental Material files. Please contact the corresponding author directly for reuse.
