# Supplementary figures and images for: Nicotinamide attenuates the virulence of colorectal cancer-associated Fusobacterium nucleatum
Source: Infect Immun. 2026 Mar 30;94(5):e00488-25. doi: 10.1128/iai.00488-25 (PMC13163194; doi:10.1128/iai.00488-25)

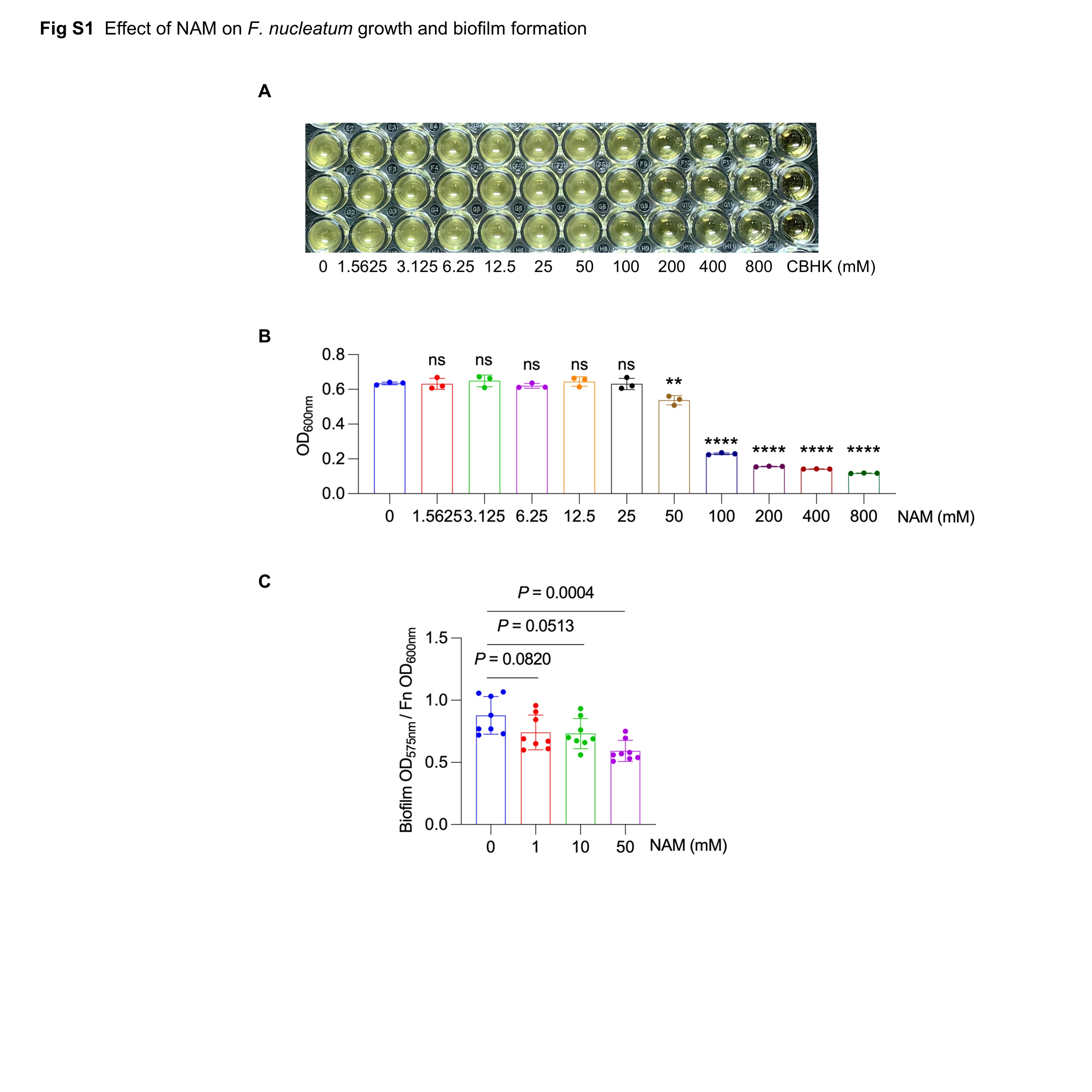

Supplement: Fig. S1 — Effect of NAM on F. nucleatum growth and biofilm formation. (A) Minimum inhibitory concentration (MIC) was performed in Columbia broth supplemented with 5 μg/mL hemin and 0.5 μg/mL menadione (CBHK). Bottom labels denote NAM concentrations and the acronym CBHK stands for blank control. (B) MIC for untreated F. nucleatum and NAM-treated F. nucleatum was quantified based on OD600 nm values. (C) Biofilm biomass was quantified by CV staining and normalized to equivalent bacterial density. The experiment was independently performed at least two times. Results are presented as mean ± SD. Each dot represents an individual sample in B and C. P values were determined by unpaired two-tailed Student’s t-test against the untreated control group in B and C (ns, P > 0.05; **P < 0.001; ****P < 0.0001). [file iai.00488-25-s0001.tiff]

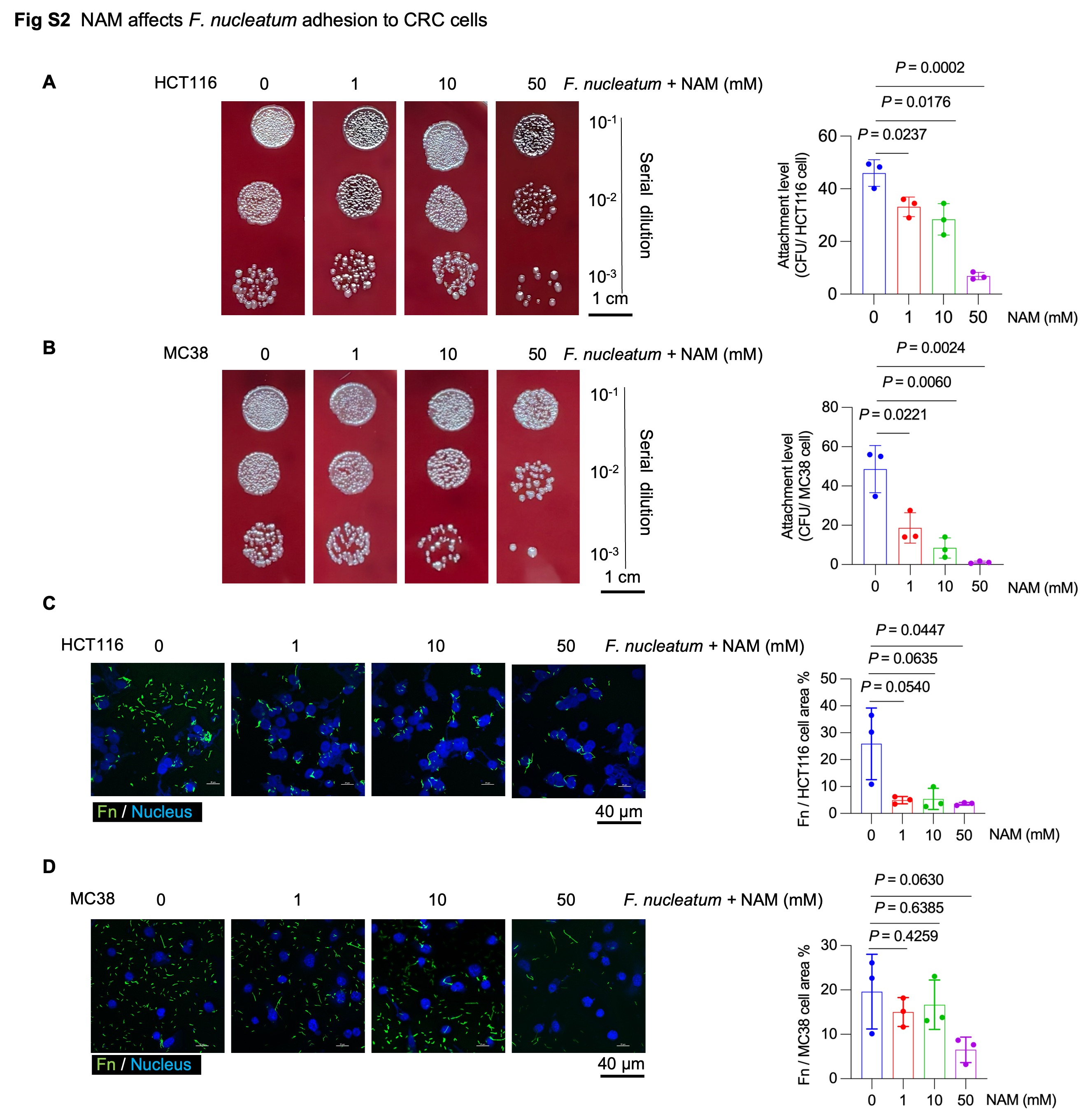

Supplement: Fig. S2 — NAM affects F. nucleatum adhesion to CRC cells.(A, B) Effect of NAM on F. nucleatum attachment to HCT116 (A) or MC38 (B) cells evaluated using CFU assay. (C, D) Representative fluorescence microscopy images showing FITC-labeled (green) F. nucleatum and DAPI-stained (blue) HCT116 (C) and MC38 (D) cells. Quantitative analysis of bacterial adhesion is shown on the right for each panel. The experiment was independently performed at least two times. Results are presented as mean ± SD. Each dot represents an individual sample in A, B, C, and D. P values were determined by unpaired two-tailed Student’s t-test in A, B, C, and D. [file iai.00488-25-s0002.jpg]

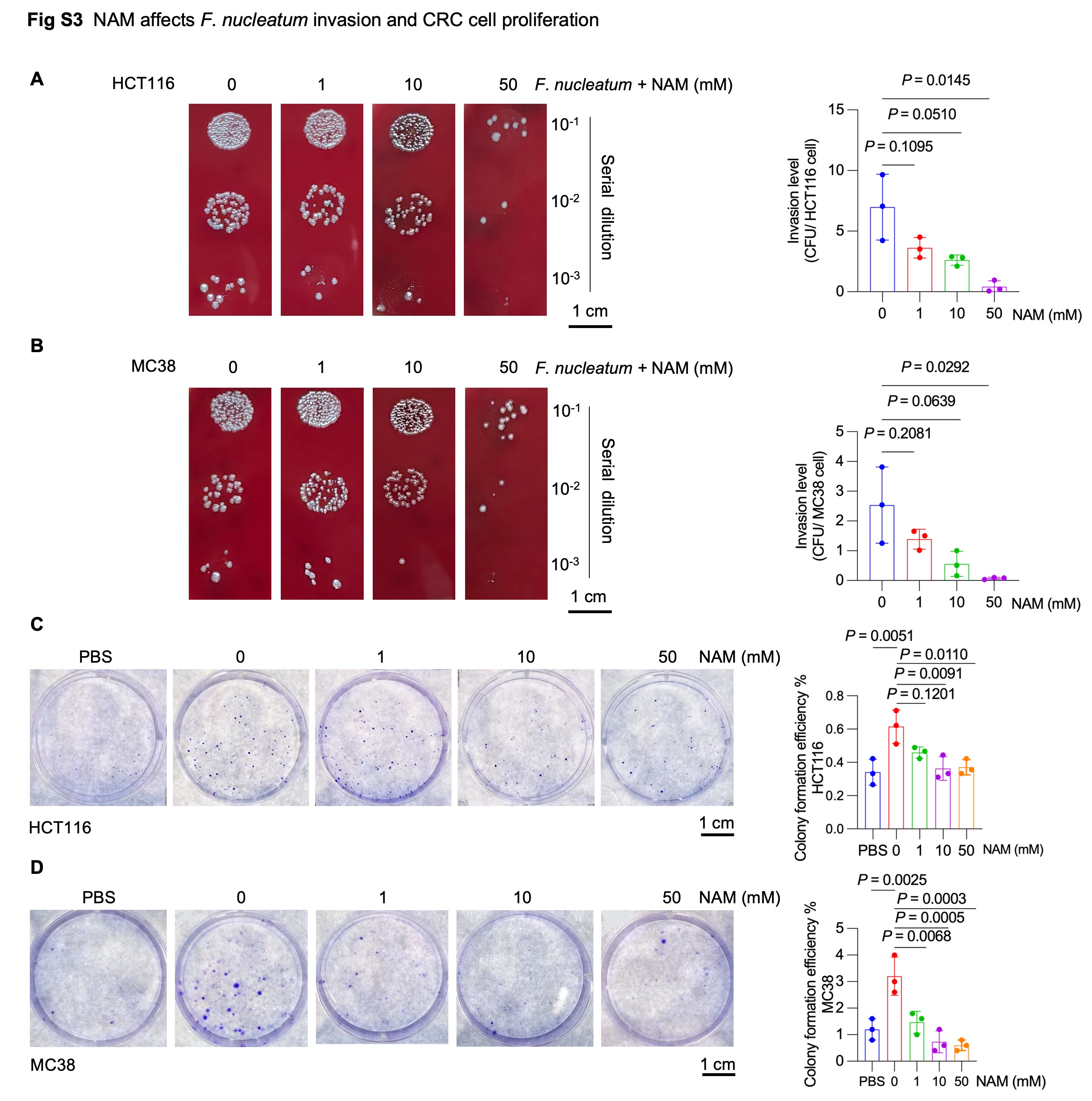

Supplement: Fig. S3 — NAM affects F. nucleatum invasion and CRC cell proliferation.(A, B) Effect of NAM on the invasion of F. nucleatum into HCT116 (A) or MC38 (B) cells evaluated using CFU assay. (C, D) Proliferation of HCT116 (C) and MC38 (D) cells in response to NAM-treated F. nucleatum were assessed using plate cloning experiment. Quantification of invading F. nucleatum and cell proliferation are shown on the right for each panel. The experiment was independently performed at least two times. Results are presented as mean ± SD. Each dot represents an individual sample in A, B, C, and D. P values were determined by unpaired two-tailed Student’s t-test in A and B, and one-way ANOVA with Tukey’s multiple comparisons test in C and D. [file iai.00488-25-s0003.jpg]
